# A novel diG motif in ORF3a protein of SARS-Cov-2 for intracellular transport

**DOI:** 10.3389/fcell.2022.1011221

**Published:** 2022-11-23

**Authors:** Ruth Cruz-Cosme, Jiantao Zhang, Dongxiao Liu, Vidhyanand Mahase, Bhargava Teja Sallapalli, Peixi Chang, Yanjin Zhang, Shaolei Teng, Richard Y. Zhao, Qiyi Tang

**Affiliations:** ^1^ Department of Microbiology, Howard University College of Medicine, Washington, DC, United States; ^2^ Department of Pathology, University of Maryland School of Medicine, Baltimore, MD, United States; ^3^ Research and Development Service, VA Maryland Health Care System, Baltimore, MD, United States; ^4^ Department of Biology, Howard University, Washington, DC, United States; ^5^ Department of Veterinary Medicine, University of Maryland, College Park, MD, United States; ^6^ Department of Microbiology and Immunology, Institute of Human Virology, Institute of Global Health, University of Maryland School of Medicine, Baltimore, MD, United States

**Keywords:** SARS-CoV-2, ORF3a, lysosome, Golgi apparatus, mutagenesis, diG motif, diG-YXXΦ interaction, intracellular transport

## Abstract

The ongoing SARS-CoV-2/COVID-19 pandemic caused a global public health crisis. Yet, everyone’s response to SARS-CoV-2 infection varies, and different viral variants confer diverse pathogenicity. Thus, it is imperative to understand how viral determinants contribute to COVID-19. Viral ORF3a protein is one of those viral determinants, as its functions are linked to induction of cell and tissues damages, disease severity and cytokine storm that is a major cause of COVID-19-related death. ORF3a is a membrane-associated protein. Upon synthesis, it is transported from endoplasmic reticulum, Golgi apparatus to plasma membrane and subcellular endomembranes including endosomes and lysosomes. However, how ORF3a is transported intracellularly remains elusive. The goal of this study was to carry out a systematic mutagenesis study to determine the structural relationship of ORF3a protein with its subcellular locations. Single amino acid (aa) and deletion mutations were generated in the putative function-relevant motifs and other regions of interest. Immunofluorescence and ImageJ analyses were used to determine and quantitate subcellular locations of ORF3a mutants in comparison with wildtype ORF3a. The wildtype ORF3a localizes predominantly (Pearson’s coefficients about 0.8) on the membranes of endosomes and lysosomes. Consistent with earlier findings, deletion of the YXXΦ motif, which is required for protein export, retained ORF3a in the Golgi apparatus. Interestingly, mutations in a double glycine (diG) region (aa 187–188) displayed a similar phenotype to the YXXΦ deletion, implicating a similar role of the diG motif in intracellular transport. Indeed, interrupting any one of the two glycine residues such as deletion of a single (dG188), both (dG187/dG188) or substitution (G188Y) of these residues led to ORF3a retention in the Golgi apparatus (Pearson’s coefficients ≥0.8). Structural analyses further suggest that the diG motif supports a type-II β-turn between the anti-parallel β4 and β5 sheets and connects to the YXXΦ motif *via* hydrogen bonds between two monomers. The diG- YXXΦ interaction forms a hand-in-hand configuration that could facilitate dimerization. Together, these observations suggest a functional role of the diG motif in intracellular transport of ORF3a.

## Introduction

The ongoing pandemic of coronavirus disease 2019 (COVID-19) by severe acute respiratory syndrome coronavirus 2 (SARS-CoV-2) has devastated many people’s lives and resulted in over one million deaths in the United States and over 6 million deaths worldwide. Due to the difference in individual’s antiviral immune response, health condition and vaccination status, each person’s experience to SARS-CoV-2 infection is different ranging from no symptom, little or mild to severe symptoms of COVID-19 and death. Therefore, it is imperative to study the underlying cause of COVID-19, and specifically which viral protein (s) contributes to the severity of COVID-19 and how an infected individual responds to this viral insult. This information will help us to design future antiviral regimens against COVID-19.

Based on the current literature, the ORF3a (Open-Reading Frame 3a) protein of SARS-CoV-2 could be one of the viral proteins that contribute to COVID-19, as it is well-known for its role in viral pathogenesis and its activities have been linked to cell death and tissue damages (Ren et al., 2020; Silvas et al., 2021; Zhang et al., 2022a; McGrath et al., 2022), induction of cytokine storm that is a major cause of COVID-19-related death (Siu et al., 2019; Xu et al., 2022) and the severity of COVID-19 (Issa et al., 2020; Majumdar and Niyogi, 2020; Lednicky et al., 2021; Nagy et al., 2021). Furthermore, ORF3a is uniquely shared by SARS-CoV and SARS-CoV-2 within the genus of β-coronaviruses (Kern et al., 2021). As these two SARS viruses cause severe human diseases and other human β-coronaviruses only cause mild human diseases, it supports the notion that ORF3a may be clinically important in causing SARS or COVID-19. For comprehensive reviews of this subject, see (McClenaghan et al., 2020; Gargan and Stevenson, 2021; Zhang et al., 2022b). Nevertheless, how exactly ORF3a contributes to the disease severity of COVID-19 is currently not well understood.

ORF3a is a membrane-associated protein that has 275 amino acids (aa) with a calculated molecular weight of 31 kD (Zhang et al., 2022b). It presents as a homodimer or tetramer (Kern et al., 2021). Each monomer has three transmembrane domains that span across the membrane and cytosol, and various functional motifs or domains that are responsible for its multifunctionalities including viral virulence, infectivity, ion channel, virus release and intracellular transport (Tan et al., 2004; Minakshi and Padhan, 2014; Issa et al., 2020; Zhang et al., 2022b). As a membrane-associated protein, ORF3a localizes on plasma membrane (Tan et al., 2004; Yuan et al., 2005; Chan et al., 2009), endosomes and lysosomes (Padhan et al., 2007; Castano-Rodriguez et al., 2018; Zhang et al., 2020; Miao et al., 2021) as well as on Golgi apparatus (Yuan et al., 2005). However, how ORF3a protein is transported to the plasma membrane and endomembranes, and what are their functional relevance to the viral life cycle and viral pathogenesis remain elusive.

One of the conserved and characteristic features of coronavirus is its subgenomic RNAs of structural and accessory proteins are produced by a replication-transcription complex (RTC) within the endoplasmic reticulum (ER)-derived and perinuclear double-membrane vesicles (DMVs) (V'Kovski et al., 2021). As ORF3a is an accessory protein, after being synthesized in ER (V'Kovski et al., 2021), it is transported to the Golgi apparatus, where it undergoes post-translational modification of O-glycosylation before it is distributed to the plasma membrane and other endomembranes such as endosomes and lysosomes (Nishimura and Balch, 1997; Oostra et al., 2006). Besides post-translational modification, a tyrosine-based sorting YXXΦ motif (where X represents any residue and Φ is a residue with a bulky hydrophobic side chain) at the cytoplasmic domain (aa 160–163) of ORF3a is required for protein sorting and transporting ORF3a from the Golgi apparatus to plasma membranes and other endomembranes such as those of endosome and lysosome (Tan et al., 2004; Minakshi and Padhan, 2014). While at lysosomes, ORF3a counteracts host cellular antiviral autophagic response by blocking the fusion of autophagosomes or amphisomes with lysosomes (Koepke et al., 2021; Miao et al., 2021).

Even though a number of well conserved functional motifs of ORF3a are linked to various functionalities (Tan et al., 2004; Lu et al., 2006; Minakshi and Padhan, 2014; Siu et al., 2019; Jin et al., 2021; Kern et al., 2021), the functional relationship of some ORF3a activities with the overall structure of ORF3a protein remains unknown. For instance, we recently showed that ORF3a induces apoptosis and necrosis through the induction of host cellular oxidative stress-mediated reactive oxygen species (ROS) production and NF-κB-mediated pro-inflammatory cytokine productions including TNFα and IL-6 (Zhang et al., 2022a), which are two strong and independent survival predictors of the patient with COVID-19 (Del Valle et al., 2020; Sayah et al., 2021). Interestingly, a single amino acid deletion at a residue G188 (∆G188) resulted in a marked increase in the ORF3a-induced cytopathic effects (Zhang et al., 2022a). The region where G188 resides is not in any one of the known functional domains. Structurally, the G188 residue is one of the two glycine residues that resides between two anti-paralleled β4 and β5 sheets that supports a type II β-turn. We surmised that this double-glycine (diG) residues could potentially be important both structurally and functionally. Therefore, we decided to carry out a mutagenesis study of ORF3a to evaluate the importance of the diG residues. In addition, we also included deletions of some of the reported functional domain and motifs (dDMs), deletions of specific cytoplasmic regions (dCRs) that span the diG region along with other single and double aa alterations that could potentially be structurally important based on our bioinformatic analysis. Therefore, the goal of this study was to establish the structural relationship of ORF3a protein with its subcellular locations that we could use to determine their functional relevance.

## Results

### SARS-CoV-2 ORF3a predominantly localizes to the membranes of endosomes and lysosomes

**FIGURE 1:**
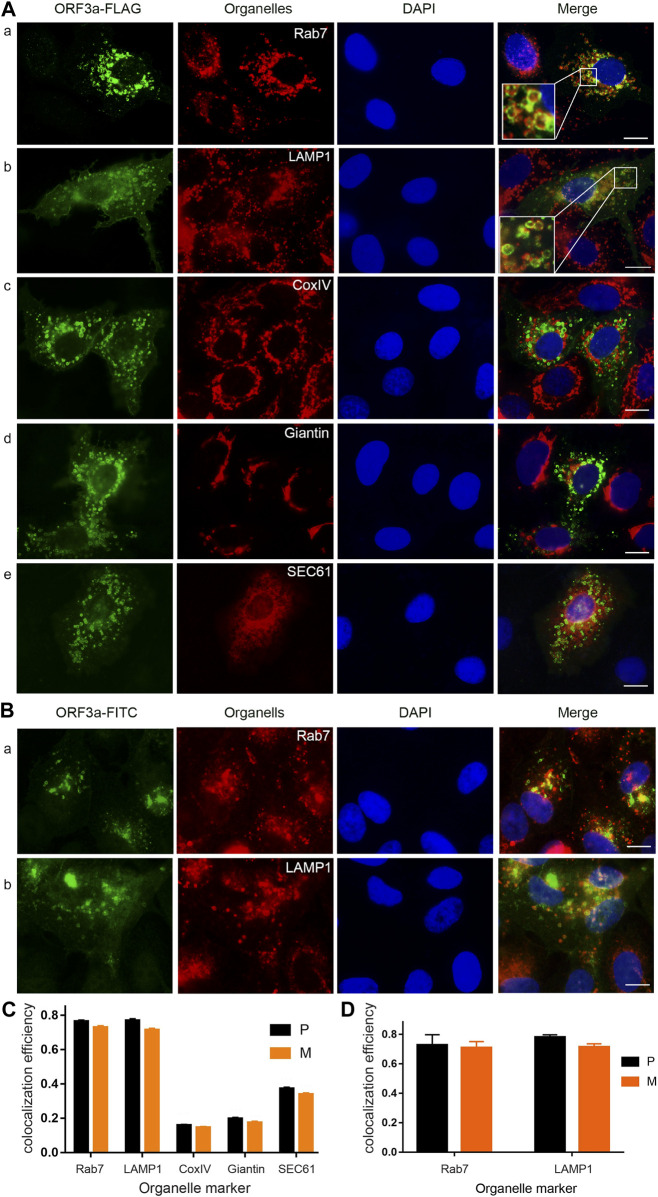
SARS-CoV-2 ORF3a protein localizes predominantly on the membranes of lysosomes and late endosomes. **(A)** Expression of *ORF3a* in the A549 cells showed abundant presence of ORF3a in late endosomes (P = 0.76 ± 0.04) as labeled by late endosome-specific marker Rab7 (a) and lysosomes (P = 0.77 ± 0.05) by LAMP-1 (b), with minor presence in mitochondria (P = 0.16 ± 0.02) by CoxIV (c), Golgi apparatus (P = 0.20 ± 0.04) by Giantin (d), and ER (P = 0.37 ± 0.04) by SEC61 (e). Enlarged image inserts in (A) and (b) show predominant association of ORF3a on the membranes of lysosomes and late endosomes. A FLAG-tagged ORF3a-carrying plasmid was transfected into A549 cells for 24 h post-transfection (*hpt*) before cells were collected for imaging. Collected cells were fixed and stained for ORF3a with anti-FLAG in green. All organelles were detected by Texas red-labeled secondary antibody in red except the ER, which was co-transfected with a pSEC61-RFP plasmid (e). Nuclei were detected by DAPI straining (blue). **(B)** Predominant localization of ORF3a in late endosomes (a) and lysosomes (b) in SARS-CoV-2 infected A549-hACE2 cells. A SARS-CoV-2 strain (USA-WA1/2020) was used to infect A549-hACE2 cells at an MOI of 0.5 for 48 h. Infected cells were fixed for IFA using anti-ORF3a (green), anti-Rab7 (a, red) and anti-LAMP1(b, red). Scale bar = 10 µm. Similar results were also seen in a different lung epithelial Calu-3 cell line (Supplementary Figure S1A). **(C)** and **(D)** Quantification of co-localization of ORF3a with different organelles as shown in **(A)** and **(B)**, respectively. To quantify the co-localization of ORF3a (green) and different organelles (red), we used ImageJ2 and JACoP plugin to analyze ORF3a co-localization with proteins of different organelles (See Materials and Methods). Both the Pearson’s correlation coefficients (P) and Mander’s overlap coefficients (M) were obtained. A total of 50 random images were used for the quantitation and the calculation of the mean and standard deviation of the P- and M-values.

Early reports showed that ORF3a inhibits the fusion of autophagosomes with lysosomes in Hep-2 and HEK293T cells (Miao et al., 2021; Zhang et al., 2021), suggesting ORF3a may associate with lysosomes. Indeed, we subsequently reported that ORF3a not only localizes on lysosomes, but it also associates with early endosomes, late endosomes, and recycling endosomes in Hep-2 cells (Zhang et al., 2020). Here, we extended our study to test subcellular localization of ORF3a in two pulmonary epithelial A549 and Calu-3 cell lines that are the primary target of SARS-CoV-2 infection. An ORF3a-FLAG plasmid that produces FLAG-tagged ORF3a were transfected into A549 or Calu-3 cells. Twenty-four hours (h) post-transfection (*hpt*), cells were fixed for immunofluorescence assay (IFA) using an anti-FLAG antibody. Organelle-specific antibodies or fluorescence RFP-tagged protein were used to detect possible co-localization of ORF3a with late endosomes (anti-Rab7), lysosomes (anti-LAMP1), Golgi (anti-Giantin), endoplasmic reticulum (ER) (SEC61-RFP) and mitochondria (anti-Cox IV). In addition, the levels of ORF3a co-localization with each one of the organelles were quantified by using ImageJ2 and JACoP and presented with Pearson’s correlation coefficients (P value) or Mander’s overlap coefficients (M value) (Bolte and Cordelieres, 2006; Zhao et al., 2022). The representative images are shown in Figure 1A with quantitation in Figure 1C for the A549 cells and Supplementary Figure S1A for Calu-3 cells.

As shown in Figure 1A, the FLAG-tagged ORF3a proteins are in green color, organelles are shown in red colors, and nuclei are in blue by DAPI staining. When these three images are merged, co-localization of ORF3a with an organelle becomes yellow in color. Otherwise, ORF3a remains in green. As shown, the ORF3a proteins are clearly associated with late endosomes (Rab7; P = 0.76 ± 0.04; M = 0.73 ± 0.04) and lysosomes (LAMP-1; P = 0.77 ± 0.06; M = 0.71 ± 0.05) as both images almost overlap entirely (Figure 1A). Note that the P values and the M values shown here represent the % of ORF3a that are correlated or overlapped with the late endosomes (76 ± 4% and 73 ± 4%) or the lysosomes (77 ± 5% and 71 ± 5%), respectively. Since these two values are similar, hereafter, we only discuss the P values. The enlarged insert images on the left bottom of the merged figures further show that ORF3a localizes primarily on the membranes of late endosomes and lysosomes. In contrast to the clear association of ORF3a with late endosomes and lysosomes, little overlaps were seen between the ORF3a and the mitochondria (Figure 1A), the Golgi apparatus (Figure 1A) and the ER (Figure 1A).

To test whether the association of ORF3a with late endosomes and lysosomes is virologically relevant, we infected A549-hACE2 cells with a SARS-CoV-2 reference viral strain USA-WA1/2020 at a multiplicity of infection (MOI) of 0.5 for 48 h. The infected cells were then fixed with 2% paraformaldehyde and permeabilized for staining with anti-ORF3a and anti-Rab7 or anti-LAMP1. Like what we showed in transfected cells (Figure 1A), the ORF3a proteins in those infected cells also localized predominantly with late endosomes (P = 0.76 ± 0.04; M = 0.73 ± 0.04) and lysosomes (P = 0.77 ± 0.05; M = 0.71 ± 0.05) (Figures 1B,D). Taken together, our data as demonstrated by producing the ORF3a protein alone or in the context of viral infection showed that ORF3a protein of SARS-CoV-2 is predominantly associated with the membranes of late endosomes and lysosomes in human pulmonary epithelial cells.

### Cytoplasmic regions of ORF3a might involve in intracellular transport of ORF3a

**FIGURE 2:**
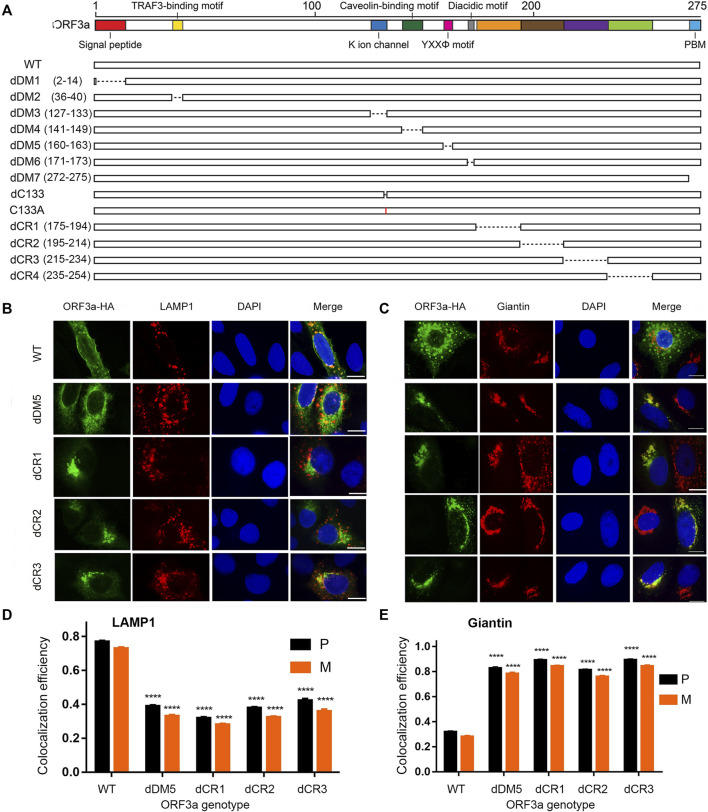
Mutagenesis of putative ORF3a functional and structural domains and corresponding changes of subcellular locations. **(A)** Schematic diagram showing where the deletions and single aa mutations were made. dDM1—dDM7 are deletions of putative functional domain motifs based on previous SARS-CoV and SARS-CoV-2 studies (Issa et al., 2020; Zhang et al., 2022b). dCR1 - dCR4, are deletions of presumably structural important cytoplasmic regions (CR) based on the bioinformatic analysis (this study). **(B)** Only those ORF3a mutants that showed different subcellular localizations from the WT are shown here. Those ORF3a mutants that show similar phenotypes to the WT are listed in Supplementary Figure S2. **(C)** ORF3a mutants show shift of subcellular localization from lysosomes to Golgi. A gene expression plasmid that carries a HA-tagged ORF3a at its C-terminal was co-transfected into A549 cells with a pRFP-LAMP1 plasmid. Transfected cells were collected 24 *hpt* and were fixed for the IFA to stain ORF3a using anti-HA antibody as shown in green. RFP, red fluorescent protein. Golgi apparatus was detected using anti-Giantin antibody as shown in red. Scale bar = 10 µm. **(D,E)** Quantification of co-localization of WT or mutated ORF3a with lysosome **(D)** or Golgi apparatus **(E)**. A total of 50 random images were used for the quantitation and the calculation of the mean and standard deviation of the P- and M-values. A pair-wise students *t*-test was used to evaluate possible statistical difference between the WT and a mutant ORF3a at the levels of significance ****p < 0.0001

Early studies including ours showed that ORF3a is a membrane-associated protein that localizes on plasma and endomembranes (Padhan et al., 2008; Zhang et al., 2020; Chen et al., 2021; Kern et al., 2021), suggesting a dynamic transport process of ORF3a within cells. To understand the possible relationship of ORF3a protein with its intracellular transport, we carried out a mutagenesis study. The overall design of the ORF3a mutagenesis panel is depicted in Figure 2A. This mutant panel was designed based on the fact that ORF3a has a number of known or predicated structural features from both SARS-CoV and SARS-CoV-2, which might link to its membrane association. We made seven deletion mutants of the domains or motifs (dDM1-7) that include deletions of the extracellular N-terminal signal peptide (aa 2–14; dDM1), a TRAF3-binding motif (aa 36–40; dDM2) (Siu et al., 2019; Jin et al., 2021), a cysteine rich domain (aa 127–133, dDM3) within the potassium (K^+^) ion channel domain (aa 81–160) (Lu et al., 2006), a caveolin-binding motifs (aa 141–149; dDM4) (Padhan et al., 2007), a YXXΦ motif (aa 160–163; dDM5), a diacidic (SGD) motif (aa 171–173; dDM6) (Tan et al., 2004; Minakshi and Padhan, 2014), and a PDZ (PSD-95/Dlg/ZO-1)-binding motif (PBM) (aa 272–275; dDM7) (Castano-Rodriguez et al., 2018; Caillet-Saguy et al., 2021).

Besides the known motifs, we also performed a computational saturation mutagenesis analysis to evaluate whether other regions of the protein or some missense mutations could affect the ORF3a location, function, or stability. Our analysis suggested that the regions in the cytoplasmic domain (CR1-CR4) could potentially alter the protein stability. Thus, we decided to generate four deletion mutants (dCR1-dCR4) where we believe those mutations could potentially render the overall protein unstable. In addition, two single aa mutations, dC133 and C133A, were also made based on a previous report that the C133 residue is required for the dimerization of the SARS-CoV ORF3a protein (Lu et al., 2006). A C-terminal HA tag was added to each ORF3a WT or mutant. The production of each ORF3a protein was confirmed by Western blot assay (Supplementary Figure S1B).

Since ORF3a predominantly associates with lysosomes as we showed in Figure 1, here, we used lysosomes as a primary endpoint to measure intracellular transport and to trace the whereabouts of the mutated ORF3a proteins. Also, as ORF3a protein is presumably synthesized in ER, after post-translational modification such as O-glycosylation (Nishimura and Balch, 1997; Oostra et al., 2006), it is translocated to the Golgi apparatus where it is transported to the plasma membrane and subcellular endomembrane such as lysosomes. Therefore, to measure intracellular transport of ORF3a, we decided to test possible co-localization of mutated ORF3a proteins between lysosomes and Golgi presumably after protein synthesis from ER (Brant et al., 2021). The same IFA method as described in Figure 1 was used to detect possible co-localization of the HA-tagged ORF3a mutants in lysosomes by anti-LAMP1 antibody and in Golgi apparatus by anti-Giantin antibody. As a result, many of the ORF3a mutants did not obviously alter their associations with lysosomes and showed similar phenotypes to the wild type (WT) ORF3a. Those images are shown in Supplementary Figure S2. Only those ORF3a mutants that showed different phenotypes from the WT are shown in Figure 2B.

Consistent with a prior report that the YXXΦ motif of SARS-CoV is involved in intracellular transport of ORF3a (Minakshi and Padhan, 2014), deletion of this motif (dDM5) indeed reduced its movement to the lysosomes (Figure 2B; P = 0.39 ± 0.04). As a result, a significant amount of the dDM5 proteins was retained at the site of Golgi apparatus (Figure 2C; P = 0.83 ± 0.05). Interestingly, a similar phenotype to the dDM5 was also seen in the dCR1-dCR3 mutants. While these mutant proteins were still seen in the lysosomes, strong co-localization of these ORF3a mutant proteins was also observed with the Golgi apparatus (Figure 2C). These observations suggest that, besides the YXXΦ motif, three additional regions at the cytoplasmic end of ORF3a as shown by the dCR1, dCR2 and dCR3 mutants might also be involved in the intracellular transport of ORF3a.

### The double glycine (diG) residues are critical for intracellular transport of ORF3a

**FIGURE 3:**
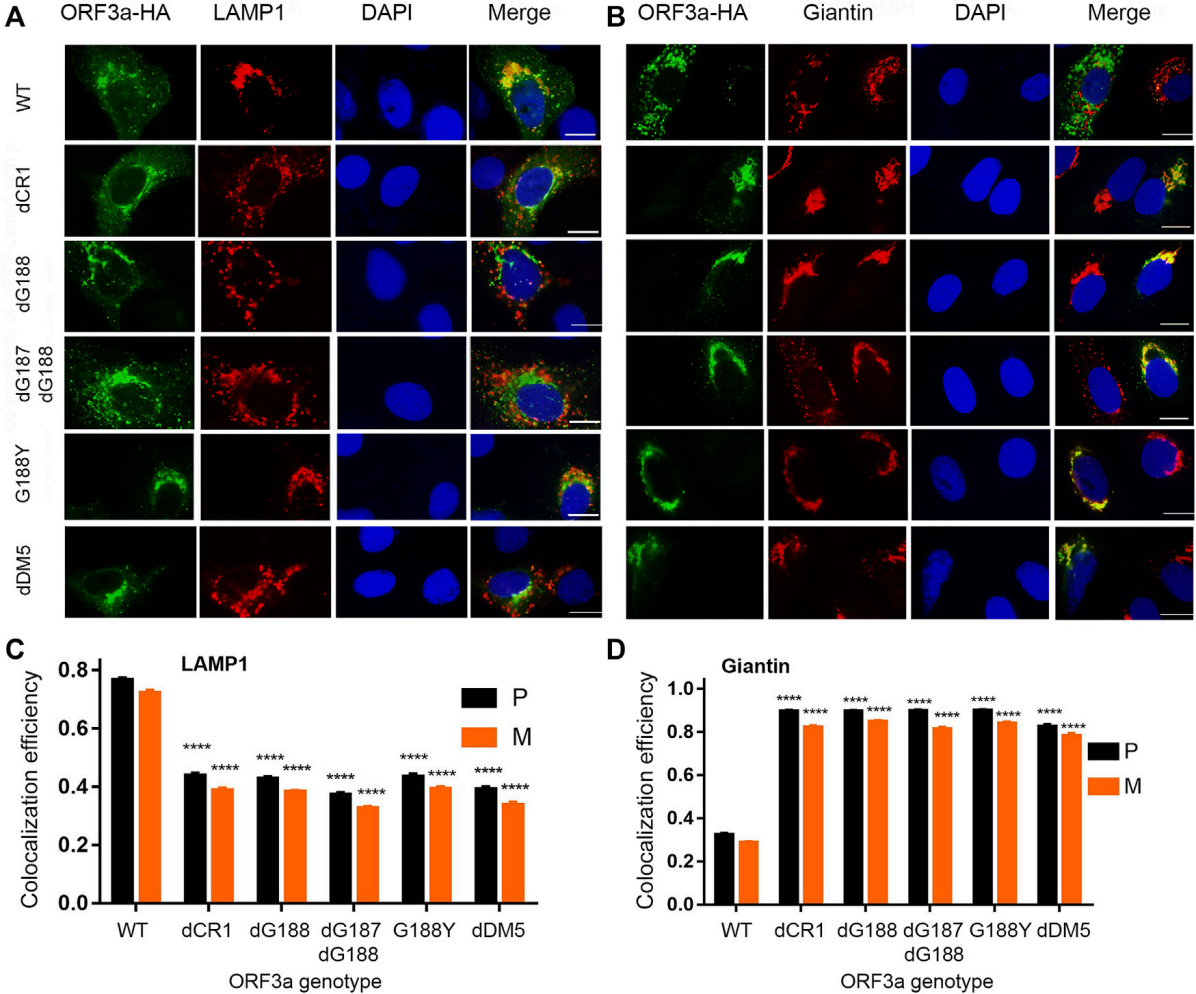
Interruption of any diG residues of ORF3a results in significant Golgi retention like deletion of the YXXΦ motif. Four diG-related ORF3a mutants were tested with the WT ORF3a and an YXXΦ motif mutant (dDM5) as controls. The four diG-related mutants include a deletion mutant of dCR1 (d175-194) that spans the diG motif and deletes the C-terminal end of the β4 sheet and N-terminal end of the β5 sheet, deletions of a single G residue (dG188) and both residues (dG187/dG188) as well as a single aa transition from glycine to tyrosine at the residue of 188 (G188Y). **(A)** Showing diminished association of the diG-related and YXXΦ motif mutant ORF3a from lysosomes, that were shown in red by anti-LAMP-1. **(B)** Showing the same diG-related and the YXXΦ motif mutations as in **(A)** resulted in increased presence in the Golgi apparatus. The Golgi apparatus was detected using anti-Giantin antibody as shown in red. Quantification of co-localization of WT or mutated ORF3a with lysosome **(C)** or Golgi apparatus **(D)** by using the same method as described in Figure 2.

Based on the ORF3a protein structural analysis (Kern et al., 2021), we found a unique diG motif between the anti-parallel β4 and β5 sheets. These two glycine residues are part of a type II β-turn that could potentially be critical in maintaining the protein structure (Zhang et al., 2022a). Since the dCR1 deletion covers the diG residues, and we previously showed that deletion of the G188 residue (dG188) significantly enhanced the cytopathic effect of ORF3a (Zhang et al., 2022a; Zhang et al., 2022b), we decided to focus on characterizing the possible role of the diG residues in intracellular transport. We hypothesized that these double glycine residues are important for intracellular transport of ORF3a from Golgi to lysosomes. To test this hypothesis, we changed these two glycine residues in three different ways, i.e., we deleted a single (dG188) and double (dG187/dG188) glycine residues, as well as converted the G188 residue glycine to tyrosine (G188Y). The results are shown in Figure 3. As a control, the WT ORF3a clearly showed its predominant presence in the lysosomes (Figure 3A; P = 0.77 ± 0.04), whereas much reduced ORF3a was seen in the Golgi compartment (Figure 3B; P = 0.32 ± 0.04). In contrast, a significant amount of the YXXΦ motif mutant protein was found co-localizing with the Golgi apparatus (P = 0.83 ± 0.05) with much reduced presence in the lysosomes (P = 0.39 ± 0.05). Like the dCR1 mutant that covers the diG motif, all three diG mutants displayed similar phenotypes to the YXXΦ mutant. Different from the dCR1, however, these three specific diG mutations showed stronger Golgi presence than in the lysosomes, suggesting this diG motif is indeed important for the transport of ORF3a from the ER-Golgi complex to lysosomes.

### Blocking Golgi export by Brefeldin A does not affect subcellular location of dG188 mutant protein

**FIGURE 4:**
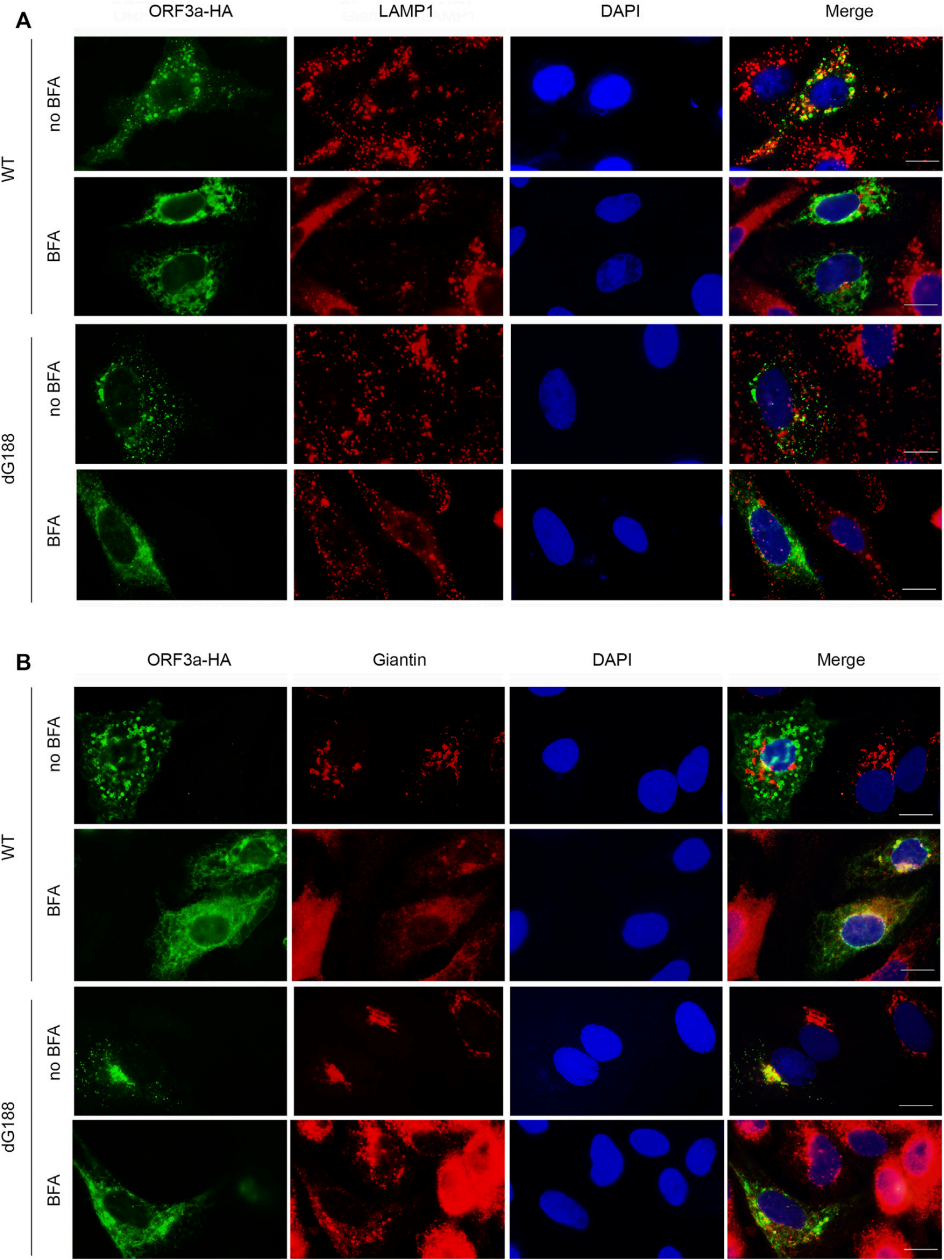
The dG188 mutant loses its ability to move out of the Golgi apparatus. The WT ORF3a or the dG188 mutant-carrying plasmid was transfected into A549 cells for 5 h. The cell cultures transfected with either plasmid was then treated with or without 1 µM Brefeldin A (BFA). Twenty hours after BFA treatment, the cells were fixed for the IFA using anti-HA antibody to stain ORF3a (green). The LAMP-1 antibody (red) was used to show lysosomes **(A)**, and the Giantin antibody (red) was used to show the Golgi apparatus **(B)**. Scale bar: 10 µm.

Since the newly discovered diG motif appears to be involved in the intracellular transport of ORF3a from Golgi to lysosomes, we next tested whether blocking the Golgi export by treating ORF3a-producing cells with an inhibitor Brefeldin A (BFA) could affect the subcellular location of the dG188 mutant protein. The dG188 mutant is chosen here because we showed earlier that this mutant significantly enhanced ORF3a-induced apoptosis and necrosis (Zhang et al., 2022a). BFA blocks Golgi export by preventing protein transportation from ER to Golgi apparatus (Helms and Rothman, 1992). The WT ORF3a-producing cells were used as a control with or without the BFA treatment. Both the WT and the dG188 *ORF3a*-expressing plasmids were transfected into A549 cells. At 5 *hpt*, culture medium was changed, and transfected cells were treated with BFA at a final concentration of 1 µM or without drug treatment (no BFA). As shown in Figure 4A, the WT ORF3a without BFA treatment localized to lysosomes as expected. However, when the same WT ORF3a-producing cells were treated with BFA, little or no ORF3a was associated with the lysosomes. Instead, much enhanced presence of the WT ORF3a was seen in the Golgi apparatus (Figure 4B), suggesting the BFA treatment successfully blocked transport of the WT ORF3a from Golgi to lysosomes. In contrast, however, the subcellular location of the dG188 mutant protein did not change with or without BFA treatment, *i.e.*, they were remained in the Golgi apparatus (Figure 4B) with little or no presence in the lysosomes (Figure 4A) regardless of the drug treatment status. Together, these results suggest that the diG motif is indeed involved in intracellular transport of ORF3a from Golgi to lysosomes.

### Possible interaction of the double glycine motif with the YXXΦ motif

**FIGURE 5:**
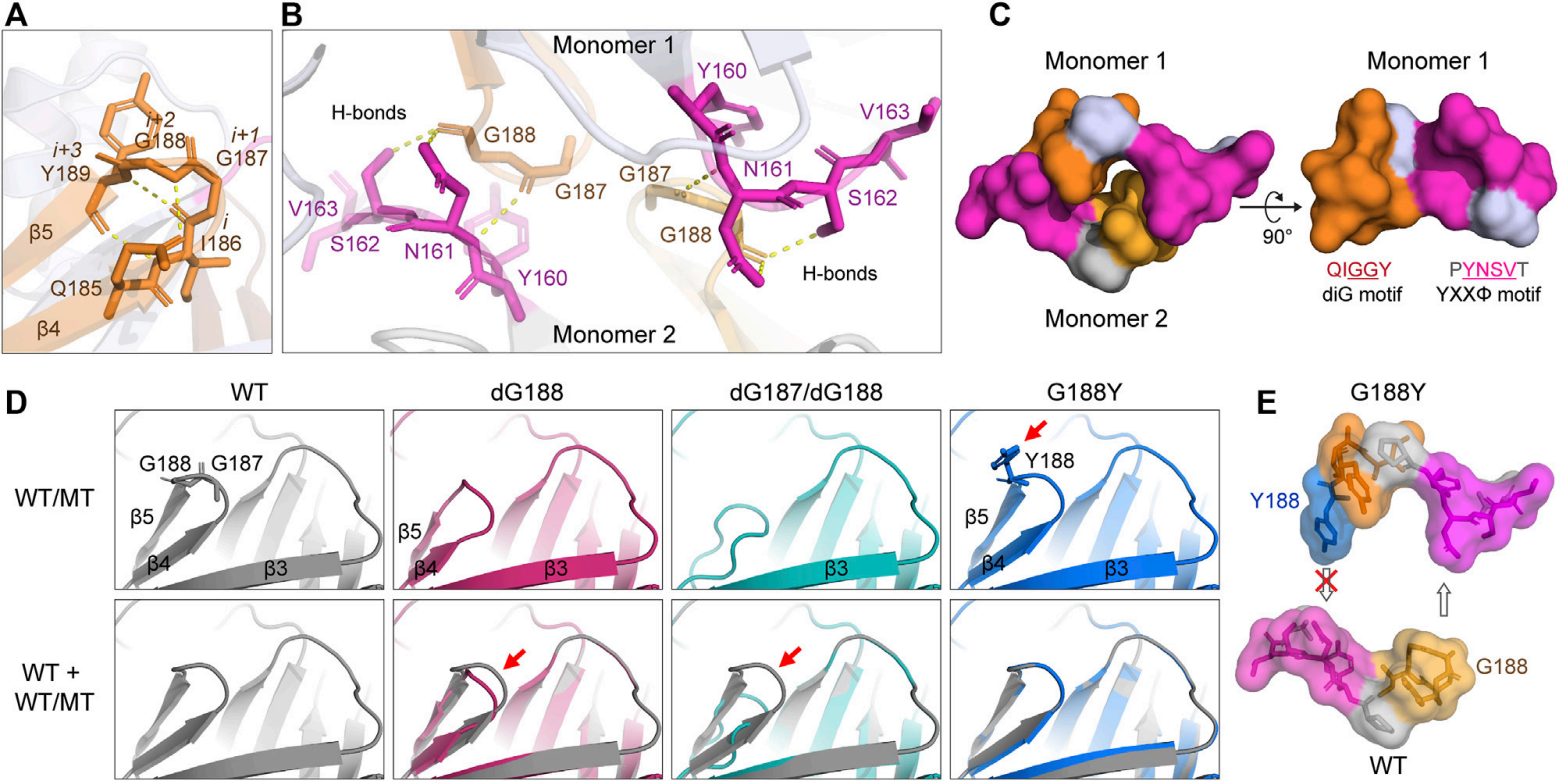
Predicated interaction of the diG motif with the YXXΦ motif. **(A)** Four residues, I186, diG motif (G187 and G188), and Y189, form a type II β-turn between β4 and β5 sheets. The hydrogen bonds were indicated with yellow dash lines. **(B)** The diG motif (G187 and G188) interacts with N161 and S162 residues of the YXXΦ motif of opposite monomer. **(C)** The diG motif and the YXXΦ motif from opposite monomers in the dimer forms a “hand-in-hand” shape structure. Only the aa 159–164 (PYNSVT) and 185–189 (QIGGY) containing YXXΦ and diG motifs (underlined) are shown. **(D)** Predicted protein structures of diG motif mutations (top panel) and their alignments with WT ORF3a (bottom panel). Red arrows indicate the major differences between WT and mutants. The protein structure was predicted using the SWISSMODEL program. 7KJR was used as the template. **(E)** The prominent aromatic side chain of Y188 blocks the formation of the “hand-in-hand” structure. All the protein 3D structures were visualized with PyMOL. All the images were prepared using Adobe Illustrator 2020.

Since both YXXΦ and diG motifs affect intracellular transport of ORF3a, we were interested in whether the diG motif has any structural relevance to the YXXΦ motif by carefully examining the diG motif position in the Cryo-EM 3D structure of ORF3a (PDB: 7KJR) (Kern et al., 2021). As illustrated in Figure 5A, diG motif forms a type II β-turn with two neighbor residues, I186 and Y189. β-turns are one of most common secondary structural motifs in protein that change the direction of polypeptide backbone. Each β-turn involves four consecutive residues either with a distance between α-carbons of *i* and *i* + 3 residues being less than 7.0 Å or forms a backbone hydrogen bond between the carbonyl of residue i (CO_*i*_) and the NH of residue *i* + 3 (NH_*i*+3_) (Hutchinson and Thornton, 1994; Koch and Klebe, 2009; Zhang et al., 2022c). Based on the *i* + 1 and *i* + 2 backbone dihedral angles, β-turns are further classified to various types, such as the most common type I and II β-turns (Zhang et al., 2022c). As for the β-turn formed by the diG motif, the distance between α-carbons of I186 (residue *i*) and Y189 (residue *i* + 3) residues is 5.3 Å, which is less than 7.0 Å. A typical intra-main chain hydrogen bond was also observed between the carbonyl of I186 and the NH of Y189. In addition, another two hydrogen bonds are formed between the side chain carbonyl of Q185 and the amide NH of G188, the amide NH of I186 and the carbonyl of Y189, to stabilize the anti-parallel β-sheets formed by β4 and β5 (Figure 5A). In short, our analysis shows that the diG motif forms a type II β-turn between the two anti-paralleled β4 and β5 sheets.

As β-turns are not only important for protein folding, but also serve as recognition structures for protein-protein interactions (Ruiz-Gomez et al., 2010). To check whether the diG motif is involved in interaction of the two monomers during dimerization of ORF3a, we searched for other residues or motifs within the ORF3a protein that could potentially interact with the diG motif. As shown in Figure 5B, two sets of diG-YXXΦ interactions are observed between monomer one and monomer two of ORF3a. Each interaction forms via three hydrogen bonds. Specifically, the carbonyl from G187 forms a hydrogen bond with the N161 backbone amide NH; the carbonyl from the G188 forms hydrogen bonds with the NH_2_ of N161 amide side chain and OH of the S162 side chain, respectively. Interestingly, within a monomeric protein, the YXXΦ motif and the diG motif are also close to each other, and together with the surrounding residues, forms a groove (aa 185–189 and aa 159–164) (Figure 5C, right panel). Therefore, two grooves from monomer one and monomer two buckle together and form a “hand-in-hand” configuration via the diG-YXXΦ interactions (Figure 5C, left panel).

Next, to see how the diG motif mutation(s) could potentially affect the diG-YXXΦ interaction as well as the “hand-in-hand” configuration, the protein structure of dG188, dG187/dG188 or G188Y was aligned with the WT ORF3a protein. The alignments of the diG motif between the WT and the mutants are shown in Figure 5D. Deletion of the G188 residue loses the G188-N161 and G188-S162 interactions, shortens the length of the β4 and β5 sheets, thus creating a new ϵ-turn, which involves three residues (aa 186–188: IGY; NH_*i*_ to CO_*i*+2_) with lower height between the β4 and the β5 (Koch and Klebe, 2009). Furthermore, deletion of both G187 and G188 residues completely abolishes the formation of the β4 and the β5 sheets as well as the β-turn, whereas the G188Y substitution remains in a similar structure as the WT. However, the prominent aromantic side chain of Y188 prevents the two monomers from reaching to each other (Figures 5D,E). Therefore, all these three diG mutations lose or block the diG-YXXΦ interactions and thus interrupt the “hand-in-hand” configuration. Since all the diG motif mutations shared similar Golgi retention phenotypes with that of the YXXΦ deletion (Figure 2B and Figure 3B), our data described here suggest a possible mechanism that the diG-YXXΦ interaction facilitates the formation of a “hand-in-hand” configuration between two monomers that is required for subcellular transport of ORF3a from the Golgi apparatus.

To further evaluate whether the diG-YXXΦ interaction is structurally conserved within SARS-CoV-2 or among β-coronaviruses, we aligned a total of 19 ORF3a protein sequences of β-coronaviruses including four SARS-CoV2 Variants of Concern (VOCs) as defined by WHO, and various other strains originated from human, bat, civet, and pangolin. These alignments showed that both diG motif and the YXXΦ motif are highly conserved among these viruses (Supplementary Figures S4A,B), suggesting the diG-YXXΦ interaction must be a highly conserved interaction within SARS-CoV-2 and among different β-coronaviruses.

## Discussion

ORF3a is a membrane-associated protein. It localizes on plasma membrane (Tan et al., 2004; Yuan et al., 2005; Chan et al., 2009), endosomes, lysosomes (Padhan et al., 2007; Castano-Rodriguez et al., 2018; Zhang et al., 2020; Miao et al., 2021), and Golgi apparatus (Yuan et al., 2005). Association of ORF3a with cell plasma membrane and subcellular endomembranes are linked to various ORF3a activities (McClenaghan et al., 2020; Gargan and Stevenson, 2021; Zhang et al., 2022b). ORF3a promotes SARS-CoV-2 viral production and virion release through lysosomal membrane-associated exocytosis pathway (Chen et al., 2021). The endosomal membrane-associated ORF3a activities may also be functionally related to clathrin-mediated endocytosis (Wong et al., 2005). When ORF3a localizes on the lysosomes, it counteracts host antiviral autophagic response by blocking the fusion of autophagosomes or amphisomes with lysosomes (Koepke et al., 2021; Miao et al., 2021). When ORF3a resides in the ER-Golgi complex, it prevents cell surface presentation of the MHC-I-viral peptide complex by reducing global trafficking of the complex and to avoid elimination by cytotoxic T cells (Arshad et al., 2022).

Although few well-conserved structural features of ORF3a protein such as the YXXΦ motif (aa 160–163) are known involving in protein sorting and intracellular transport of ORF3a from Golgi to plasma membranes (Tan et al., 2004; Minakshi and Padhan, 2014), the relationship of the overall protein structure with the whereabouts of ORF3a proteins, and how ORF3a proteins are transported within cells remain elusive. In this study, we carried out a systematic mutagenesis study of ORF3a protein with a goal to decipher the association of ORF3a protein structure with their subcellular locations. Seven deletion mutants (dDM1-dDM7) were generated in the regions where either known functional motifs or structurally important regions reside. Four cytoplasmic deletion mutants (dCR1-dCR4) were also made based on our structural and bioinformatic analyses (Figure 2A). To establish a baseline control, we first showed in lung epithelial A549 and Calu-3 cell lines that ORF3a localizes predominantly on the membranes of lysosomes with a Pearson’s correlation coefficient of 0.77 ± 0.05 with minor presence in other organelle compartments such as Golgi with a Pearson’s correlation coefficient of 0.20 ± 0.04 (Figure 1, Supplementary Figure S1A). The associations of virus-born ORF3a with endosomes and lysosomes were further confirmed in the context of SARS- CoV-2 infection (Figure 1B). These results are consistent with previous reports (Yuan et al., 2005; Padhan et al., 2007; Castano-Rodriguez et al., 2018; Zhang et al., 2020; Miao et al., 2021).

A conserved feature of β-coronaviruses is that subgenomic RNA of *ORF3a* along with other accessory and structural viral proteins are produced by an RTC (Angelini et al., 2013; Hagemeijer et al., 2014; Oudshoorn et al., 2017), which binds to the ER membrane-derived DMVs. After ORF3a protein is synthesized in ER, it is exported from ER to Golgi apparatus, where it undergoes post-translational modification of O-glycosylation before being exported to various destinations within cells including lysosomes and plasma membrane (Nishimura and Balch, 1997; Oostra et al., 2006). Here, we used lysosomes and Golgi as the endpoints to measure how the ORF3a structural mutations affect intracellular transport of ORF3a from the ER-Golgi complex to rest of the subcellular organelles.

Our results showed that all the seven dDM deletion mutants except dDM5 showed similar phenotypes as the WT ORF3a and predominantly associated with lysosomes, suggesting deletions in these protein regions have no clear impact on the ability of these proteins to be exported from the ER-Golgi complex to lysosomes (Supplementary Figure S2). In contrast, deletion of the YXXΦ motif (dDM5) resulted in retention of ORF3a in the Golgi apparatus (Figure 2B). The YXXΦ motif, residing at aa 160–163 of ORF3a, is a highly conserved tyrosine-based protein sorting motif that is known involving in protein sorting and intracellular transport of proteins of various viruses (Windheim et al., 2004). Like in other viruses, deletion of the YXXΦ motif in SARS-CoV-2 abolished its ability of intracellular transport movement and retained it in the Golgi compartment (Minakshi and Padhan, 2014).

Like the mutational effect of the YXXΦ motif, three of the cytoplasmic deletions (dCR1-dCR3) also resulted in retention of the mutant ORF3a in the Golgi compartment (Figure 2B). Since these three deletions are in the cytoplasmic domain of ORF3a spanning from the β4 sheet to the β8 sheet, these results implicate a possible importance of the cytoplasmic domain of ORF3a in intracellular transport. Conversely, the C-terminal end of ORF3a might not be involved in the intracellular transport of ORF3a, as neither the dDM7, a deletion of a DMZ-binding motif (∆aa272-275), nor the dCR4 mutant that interrupts a highly conserved C-terminal region at aa 235–254, had any effect on the lysosomal association of ORF3a (Supplementary Figure S2).

Upon analysis of the 3D-protein structure of ORF3a (PDB: 7KJR) (Kern et al., 2021) and alignments of 19 ORF3a protein sequences from various β-coronaviruses, we showed that the diG motif is highly conserved (Figure 5, Supplementary Figure S4). Our analysis further showed that this diG motif forms a type II β-turn that resides between the anti-parallel β4 and β5 sheets, which could potentially be structurally important and affect the subcellular location of ORF3a (Zhang et al., 2022a; Zhang et al., 2022c). Indeed, interrupting any one of the two glycine residues such as deleting one residue (dG188), both residues (dG187 and dG188) or altering one residue (G188Y) all resulted in the retention of ORF3a in the Golgi apparatus (Figure 3). Blocking transport of ORF3a from Golgi to lysosomes by BFA treatment further showed that it indeed prevented the Golgi export of the WT ORF3a, but it did not have any clear effect on the dG188 mutant (Figure 4). Therefore, these data suggest the diG motif is necessary for intracellular transport of ORF3a from Golgi to lysosomes.

While our current effort is to test the functional relevance of these described mutants to viral infection, our earlier comparison of the WT ORF3a with one of the described dG188 mutants in a functional study showed that the dG188 mutant elicits much stronger host cellular oxidative stress and pro-inflammatory immune responses than the WT ORF3a that resulted in a marked increase in the ORF3a-induced apoptotic cell death (Zhang et al., 2022a). As ORF3a displays different activities when it resides on the lysosomes vs. the ER-Golgi complex (Yuan et al., 2005; Miao et al., 2021), and natural diG mutant variants such as the G188 mutations are also found in the emerging viruses (from the GISAID database), these findings suggest that some of the natural SARS-CoV-2 variants could potentially exert more severe cytopathic effects to host cells than the original virus. Our future experiments will focus on characterizing the ORF3a mutants described here and natural mutant variants that block Golgi export and further assess their functional consequences to ORF3a-specific activities. Overall, our observations collectively suggest that we have discovered a novel diG motif that is critical not only for intracellular trafficking of ORF3a from Golgi, but also has significant functional impact on ORF3a-medicated cytopathic effects on host cells.

Although double-glycine residues should be commonly found in protein sequences, to the best of our knowledge, there is no prior report showing the double-glycine residues could serve as a structurally-import motif or played an important functional role in a viral protein. However, a functional double-glycine motif (*aka* GG-motif) has been reported in Gram-positive and Gram-negative bacteria (Dirix et al., 2004a; Dirix et al., 2004b). These GG-motifs serve as N-terminal leader peptides in class II bacteriocins where they are cleaved off by the peptidase C39 domain of an ABC transporter protein, which result in secretion and subsequent translocation of the bacteriocins across the cytoplasmic membrane (van Belkum et al., 1997; Bobeica et al., 2019). Similar to bacterial GG motif, the diG motif in ORF3a also involves in translocation, but it is not known whether the diG motif of ORF3a is subject to enzymatic cleavages. Nevertheless, we show here, for the first time, that a diG motif plays an important role in intracellular transport of a viral protein.

From the perspective or ORF3a protein structure, our structural analysis of a Cryo-EM model of ORF3a protein showed interactions between the diG motif and the YXXΦ motif between opposite monomers *via* hydrogen bonds (Figure 5B). As the result, two grooves formed by the diG motif and the YXXΦ motif buckle together forming a “hand-in-hand” configuration (Figure 5C), suggesting this diG-YXXΦ interaction could facilitate the dimerization of the ORF3a. Note that the C133 residue was previously shown to be required for the dimerization of SARS-CoV ORF3a protein, and the dC133 and C133A mutations interrupt dimerization (Lu et al., 2006). In this study, we also tested these two mutants with the assumption that they may affect dimerization and subcellular locations of SARS-CoV-2 ORF3a. However, neither C133 nor C133A mutant altered subcellular locations, and both showed similar phenotypes as the WT ORF3a (Supplementary Figure S3, S4C). These two mutants along with mutants to interrupt the diG-YXXΦ interaction will be further tested for their effects on dimerization in the future.

The presumptive diG-YXXΦ interaction and the “hand-in-hand” configuration could certainly explain, in the context of protein structure, some of the mutational effects we observed in the diG mutations. Specifically, interruption of any one of the two glycine residues such as deleting a single residue (dG188), both residues (dG187 and dG188) or altering one residue (G188Y) will interrupt the diG-YXXΦ interaction and block the “hand-in-hand” structure formation (Figures 5D,E). As consequences, conceivably, all these three mutations might prevent or weaken protein dimerization of ORF3a. While we do not have direct experimental evidence to support these predictions, this possibility will certainly be tested in the future. Nonetheless, the structural implication of the diG motif as shown by these mutants are consistent with the idea that the diG-YXXΦ interaction is critical for intracellular transport of ORF3a from Golgi apparatus to rest of the subcellular organelles, as our observations certainly showed that all these mutations were retained in the Golgi compartment like the mutation of the YXXΦ motif (Figure 3).

As our data also suggest the dCR2 and dCR3 mutants might also be involved in the intracellular transport of ORF3a (Figure 2B), interruption of ORF3a protein structure at these cytoplasmic domains may also affect ORF3a dimerization. For instance, when ORF3a is in a dimmer formation, the inner β-sheets, β3 and β8, from each monomer form a strong and stable link through a large and highly complementary interface along with a continuous hydrophobic core (Supplementary Figure S4C) (Kern et al., 2021). In the dCR3 mutant (aa 215–234), β8 sheet was deleted, leading to the abolishment of the hydrophobic core formation. In the dCR2 mutant (aa 195–214), the outer β-sheets, β6 and partial β7, which connect inner β-sheets, β5 and β8, were deleted, affecting the inner β-sheets formation and/or the stability of the overall protein structure. This premise is consistent with the result of our bioinformatic analysis. In contrast, the deletion generated outside the cytoplasmic domain as shown by the dCR4 mutant (aa 235–254), does not affect the β-sheets, nor did it affect intracellular transport of ORF3a (Supplementary Figure S2).

In summary, we systematically investigated the structural relationship of ORF3a protein with its ability to transport from the ER-Golgi complex to lysosomes through a mutagenesis study. Besides the YXXΦ motif that was already known for its role in intracellular protein trafficking, we uncovered a novel diG motif that is also critical for intracellular transport. In addition, we showed that diG motif supports a type II β-turn between the β4 and the β5 sheets of ORF3a and interacts with the YXXΦ motif possibly to promote protein dimerization and protein trafficking within cells.

## Materials and methods

### Cell line, virus, and culture

Lung epithelial cell lines Calu-3 (NIH/NIAID, NR-55340) and A549-ACE2 (NIH/NIAID, NR-53821) were obtained from BEI resources (https://www.beiresources.org/). A549 (ATCC^®^ CCL-185™) and HEK 293T (ATCC^®^ CRL-1573™) cells were purchased from ATCC. All cells were maintained in Dulbecco’s modified Eagle’s medium (DMEM) supplemented with 10% fetal calf serum (FCS) and penicillin (100 IU/ml)-streptomycin (100 μg/ml) and amphotericin B (2.5 μg/ml) (de Bruyn Kops and Knipe, 1988). A SARS-CoV-2 reference viral strain USA-WA1/2020 (Genbank accession number: MN985325) was used in this study. For virus infection, the cells were plated on coverslips in a 12 or 24-well cell culture plate and grew overnight to 90% confluency. The cell culture plate was then moved to a biosafety level-3 (BSL-3) suite where the viral infection was carried out. The virus was diluted to the desired MOI and added to the cells followed by incubation for the desired length of time before being fixed for further analysis.

### Molecular cloning and mutagenesis of SARS-CoV-2 ORF3a mutants

A gene expression plasmid that produces a FLAG-tagged WT ORF3a at its C-terminus (pCAG-nCoV-ORF3a-FLAG) was described previously (Zhang et al., 2020). To replace the FLAG tag of ORF3a with a HA tag, the DNA insert of *ORF3a*-*HA* was generated by PCR with a pair of primers (nCoV-ORF3a-HA-F and nCoV-ORF3a-HA-R) by using the pCAG-nCoV-ORF3a-FLAG as a template. The amplified PCR insert was gel-purified and digested with the same *XhoI* and *AgeI* restriction enzymes as they were used to prepare for the pCAG vector. After the ligation of the vector and the insert at the *XhoI* and *AgeI* sites, a new plasmid that produces the HA-tagged WT ORF3a (pCAG-nCoV-ORF3a-HA) was made.

For the ORF3a mutagenesis, the plasmid pCAG-nCoV-ORF3a-HA that carries a WT *ORF3a* was used as a template to generate the respective ORF3a mutants as shown in Figure 2A using an overlapping PCR method (Cruz-Cosme et al., 2020). Specifically, to generate the pCAG-nCoV-ORF3a-dDM2-HA plasmid that carries the domain two deletion (dDM2), two overlapping PCR fragments were made with two primer pairs: the primer pair 1: nCoV-ORF3a-HA-F and ORF3-HA-d36-40-up, and the primer pair 2: ORF3-HA-dDM2-down and nCoV-ORF3a-HA-R. The same overlapping PCR method was used to generate all the other ORF3a deletion mutant (dDM1-dDM7; dCR1-dCR4) and single aa changes as shown in Figure 2A. The accuracy of all the ORF3a mutants generated as described were verified by Sanger DNA sequencing. All the nucleotide primers that were used in the generation of the *ORF3a* mutant-carrying plasmids are listed in Supplementary Table 1.

### Plasmid transfection

To examine subcellular location of the wild type and ORF3a mutant proteins, the *ORF3a*-carrying plasmids were co-transfected with one of the following plasmids that expresses an organelle-specific marker, *i.e*., the pMch-sec61-beta plasmid for the detection of ER and the ER-Golgi intermediate compartment (Addgene Cat# 49155) (Zurek et al., 2011); the pLamp1-RFP plasmid for the detection of lysosomes (Addgene Cat# 1817) (Sherer et al., 2003), and the pRFP-Rab7 plasmid for the detection of late endosome (Addgene Cat# 14436). These plasmids were purchased from Addgene (http://www.addgene.org). The transfection reagent (Lipofectamine 3,000) was purchased from Invitrogen and used according to the manufacturer’s protocol.

### Antibodies and reagents

Mouse antibodies against Tubulin (4G1, sc-58666), late endosomes (Rab7, B-3, sc-376362), and LAMP1 (E-5, sc-17768) were purchased from Santa Cruz Biotechnology (Santa Cruz, CA). Mouse anti-FLAG (M2), and rabbit anti-HA (H6908) antibodies were purchased from Sigma (St. Louis, MO). Rabbit anti-FLAG (PA1-984B) was purchased from Invitrogen (Carlsbad, CA). Rabbit anti-Giantin (ab80864), and anti-CoxIV (ab16056) were purchased from Abcam (Boston, MA). Rabbit anti-ORF3a (LS-C829863) was from LS Bio (Seattle, WA). Mouse anti-ORF3a (MAB10706) was bought from R&D systems (Minneapolis, MN). Brefeldin A (BFA) was purchased from Sigma Aldrich (Cat# B7651) and dissolved in DMSO at a concentration of 5 mg/ml and stored at −20°C.

### Immunofluorescence Assay (IFA)

Cells grown on coverslips were fixed with 1% paraformaldehyde for 10 min (min) at room temperature and permeabilized in 0.2% Triton for 20 min on ice. Immunostaining was performed by sequential incubation with primary antibodies and Texas red (TR)-labeled secondary antibodies (Vector Laboratories, Burlingame, Calif.) for 30 min each (all solutions in PBS). Washing steps using PBS were performed after each incubation with paraformaldehyde, Triton, or antibodies, after antibody incubation. Finally, cells were equilibrated in PBS, stained for DNA with DAPI (0.5 μg/ml), and mounted with Fluoromount G (Fisher Scientific, Newark, Del.).

### Confocal microscopy

Cells were examined with a Leica TCS SPII confocal laser scanning system. Two or three channels were recorded simultaneously and/or sequentially and controlled for possible breakthrough between the fluorescein isothiocyanate and Texas Red signals and between the blue and red channels.

### Quantification of the co-localization of ORF3a with subcellular organelles

An image software ImageJ2 image and JACoP plugin (https://imagej.net) were used for quantification of co-localization of ORF3a with different organelle biomarkers (Bolte and Cordelieres, 2006; Zhao et al., 2022). The image analysis process is shown in Supplementary Figure S1C by using Figure 1A as an example. Briefly, the merged image of selected ORF3a-positive cell was split into red and green channels using the color function under image menu. Then the JACoP plugin was used to analyze protein co-localization. The default threshold of red channel (showing the organelle biomarker protein such as lysosomes detected by anti-LAMP1) was used. The threshold of green channel (ORF3a) was set to 50 for consistency. After analysis, the Pearson’s correlation coefficient (*p*) and Mander’s overlap coefficient (M) were obtained for comparative degree of co-localization of ORF3a and organelle biomarker proteins. A total of 50 random images were used for the analyses and the calculation of the mean and standard deviation of the P- and M-values. A pair-wise student *t* test was used to evaluate possible statistical difference between the WT and a mutant ORF3a at the levels of significance ****p < 0.0001.

### Protein Structure Analysis and Protein Sequence Alignment

High resolution of ORF3a protein 3D structure was obtained from RCSB PDB (7KJR; 2.08 Å). PyMOL was exploited for the residue-residue interaction analysis. Protein structure modeling was performed using SWISS-MODEL (swissmodel.expasy.org). 7KJR was used as the template. All ORF3a proteins sequences were obtained from NCBI (www.ncbi.nlm.nih.gov). The respective accession numbers were indicated in Supplementary Figure S4A. Protein amino acid sequence alignment was performed using MEGA 11. ClustalW method and default setting were used. The sequence similarity and secondary structure information were indicated using ESPrint 3.0 (https://espript.ibcp.fr). All figures were prepared using PyMOL and Adobe Illustrator 2020.

## Data Availability

The original contributions presented in the study are included in the article/Supplementary Material, further inquiries can be directed to the corresponding authors.
