# Supplementary material for: A novel diG motif in ORF3a protein of SARS-Cov-2 for intracellular transport
Source: Front Cell Dev Biol. 2022 Nov 23;10:1011221. doi: 10.3389/fcell.2022.1011221 (PMC9727819; doi:10.3389/fcell.2022.1011221)
Supplement: Supplementary file 1 [file Table1.docx]

**Table 1 Oligonucleotide Primers for the molecular cloning and mutation of SARS-CoV-2 ORF3a**

ORF3a-HA-F ccgCTCGAGGCCACCATGGATTTG

ORF3a-HA-R ccgACCGGTTTAGGCGTAGTCAGGCACGTCGTAAGGATACAAAGGCACGCTAGTAGTCGTCGT

ORF3a-HA-dDM1-F ccgCTCGAGGCCACCATG AAGCAAGGTGAAATCAAGGATGCTAC

ORF3a-HA-dDM2-up AGCCATCCGAAAGGGAG TATCGTTGCAGTAGCGCGAACAA

ORF3a-HA-dDM2-down CGCTACTGCAACGATA CTCCCTTTCGGATGGCTTATTGTT

ORF3a-HA-dDDM3-up ATGGGTTTTTGGAACGCCTCATTATTATTCTTACAAAGTTTATACTCTG

ORF3a-HA-dDM3-down AGAATAATAATGAGG CGTTCCAAAAACCCATTACTTTATGATG

ORF3a-HA-dDM4-up CGTAACAATTAGTATGAAGTAATGGGTTTTTGGAACGGCA

ORF3a-HA-dDM4-down CAAAAACCCATTACTT CATACTAATTGTTACGACTATTGTATACCTTAC

ORF3a-HA-dDM5-up TGACAATTGAAGAAGT AGGTATACAATAGTCGTAACAATTAGTATGC

ORF3a-HA-dDM5-down CGACTATTGTATACCT ACTTCTTCAATTGTCATTACTTCAGGTG

ORF3a-HA-dDM6-up TAGGACTTGTTGTGCC AGTAATGACAATTGAAGAAGTTACACTATTGTAAG

ORF3a-HA-dDM6-down TTCAATTGTCATTACT GGCACAACAAGTCCTATTTCTGAACAT

ORF3a-HA-dCR1-up AACACAGTCTTTTACTCCAGA GCCATCACCTGAAGTAATGACAATTGAAGA

ORF3a-HA-dCR1-down GTCATTACTTCAGGTGATGGC TCTGGAGTAAAAGACTGTGTTGTATTACAC

ORF3a-HA-dCR2-up TGTACTCAATTGAGTTGAGTA TTCCCATTTTTCAGTATAACCACCAATCTG

ORF3a-HA-dCR2-down GGTTATACTGAAAAATGGGAA TACTCAACTCAATTGAGTACAGACACTGGTGTT

ORF3a-HA-dCR3-up TTCAGGCTCATCAACAATTTT CAGCTGGTAATAGTCTGAAGTGAAGTAACTGTG

ORF3a-HA-dCR3-down ACTTCAGACTATTACCAGCTG AAAATTGTTGATGAGCCTGAAGAACATGTC

ORF3a-HA-dCR4-up CATTACTGGATTAACAAC ATTGTAGATGAAGAAGGTAACATGTTCAAC

ORF3a-HA-dCR4-down GTTACCTTCTTCATCTACAAT GTTGTTAATCCAGTAATGGAACCAATTTATGAT

ORF3a-HA-dG188-down GAACATGACTACCAGATTGGT TATACTGAAAAATGGGAATCTGGAGT

ORF3a-HA-dG188-up ATTCCCATTTTTCTGTATA ACCAATCTGGTAGTCATGTTCAGAAAT

ORF3a-HA-G188Y-down ATTTCTGAACATGACTACCAGATTGGTTAT TATACTGAAAAATGGGAATCTGGAGT

ORF3a-HA-G188Y-up ATTCCCATTTTTCTGTATA ATAACCAATCTGGTAGTCATGTTCAGAAAT

cov2-ORF3a-HA-dG187dG188-down GAA CAT GAC TAC CAG ATT TAT ACT GAA AAA TGG GAA

cov2-ORF3a-HA-dG187dG188-down TTC CCA TTT TTC AGT ATA AAT CTG GTA GTC ATG TTC

cov2-ORF3a-HA-dC133-down CTT TGG CTT TGC TGG AAA CGT TCC AAA AAC CCA TTA CTT

cov2-ORF3a-HA-dC133-up TGG GTT TTT GGA ACG TTT CCA GCA AAG CCA AAG CCT CAT

cov2-ORF3a-HA-C133A-down CTT TGG CTT TGC TGG AAA GCC CGT TCC AAA AAC CCA TTA CTT

cov2-ORF3a-HA-C133A-up TGG GTT TTT GGA ACG GGC TTT CCA GCA AAG CCA AAG CCT CAT
