# Supplementary figures and images for: A novel diG motif in ORF3a protein of SARS-Cov-2 for intracellular transport
Source: Front Cell Dev Biol. 2022 Nov 23;10:1011221. doi: 10.3389/fcell.2022.1011221 (PMC9727819; doi:10.3389/fcell.2022.1011221)

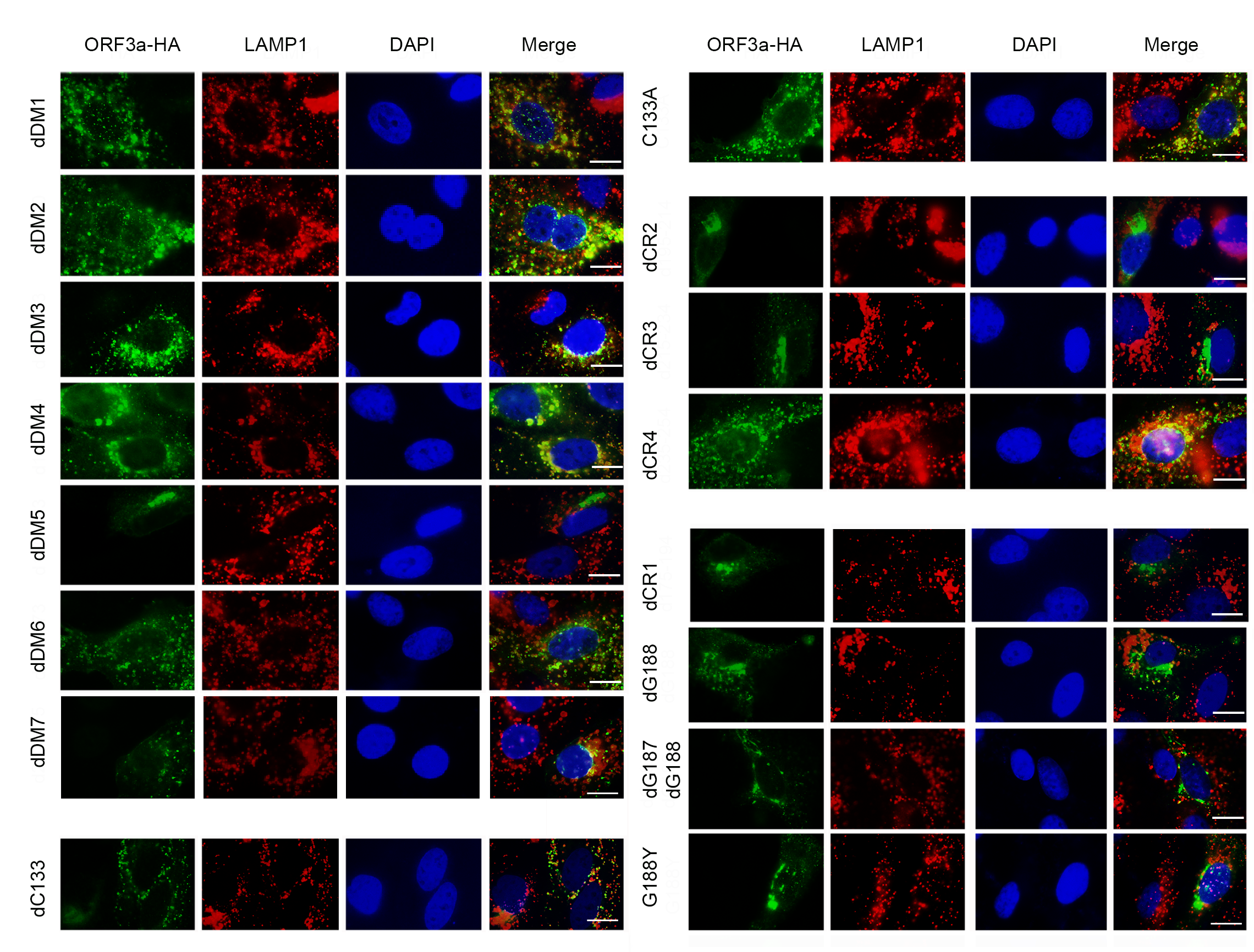

Supplement: Supplementary file 2 [file Image3.TIF]

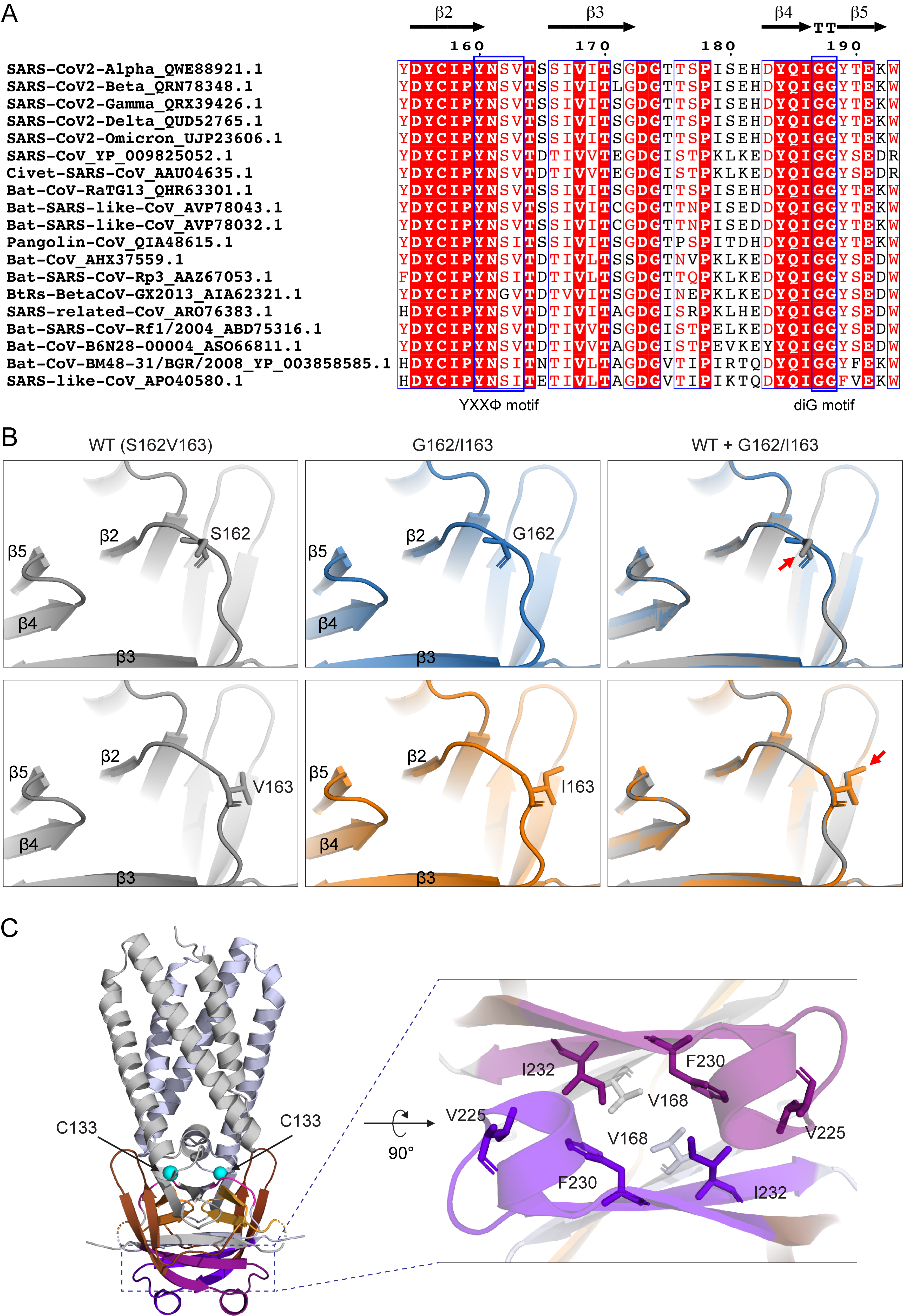

Supplement: Supplementary file 3 [file Image4.TIF]

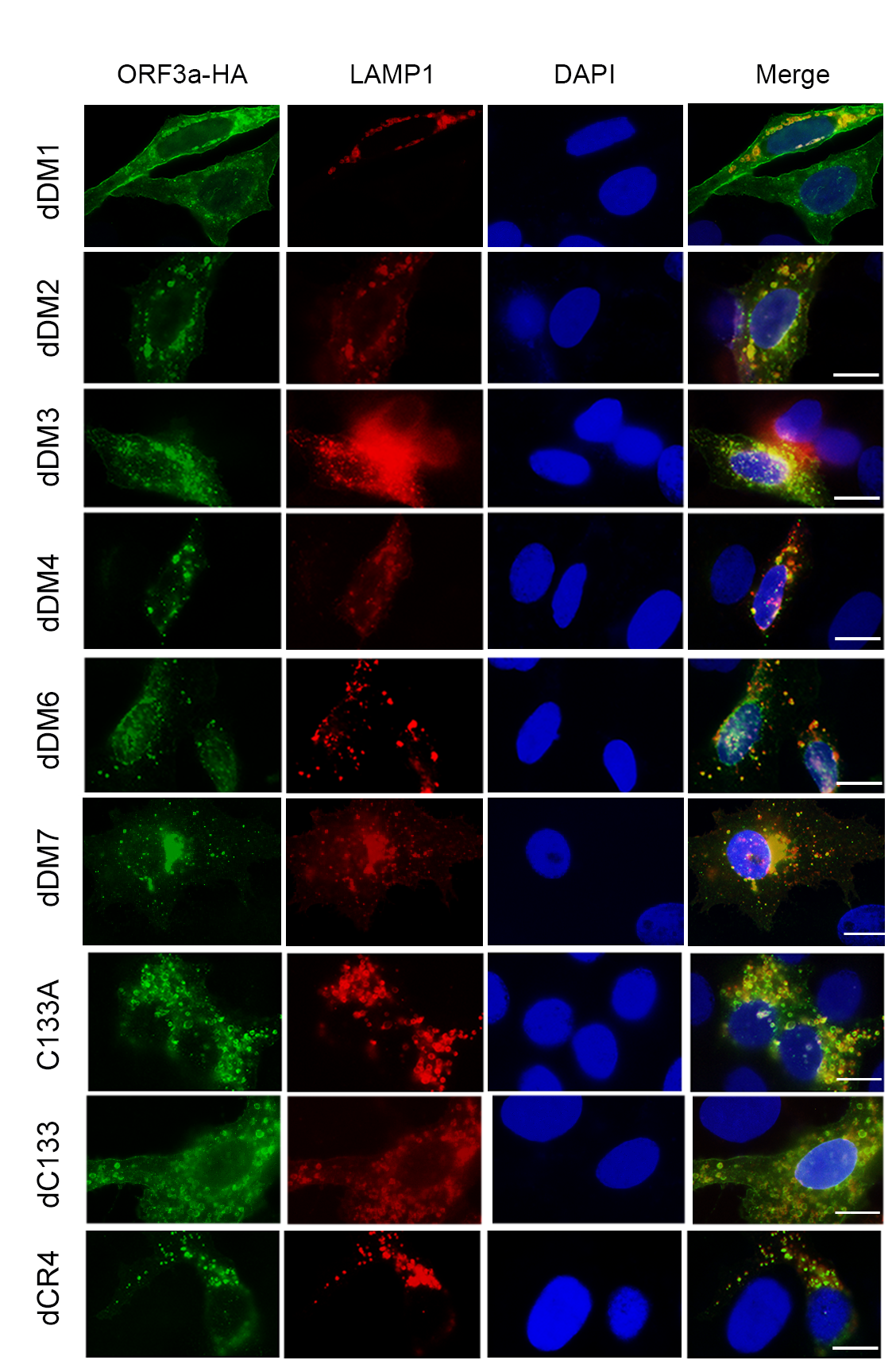

Supplement: Supplementary file 4 [file Image2.TIF]

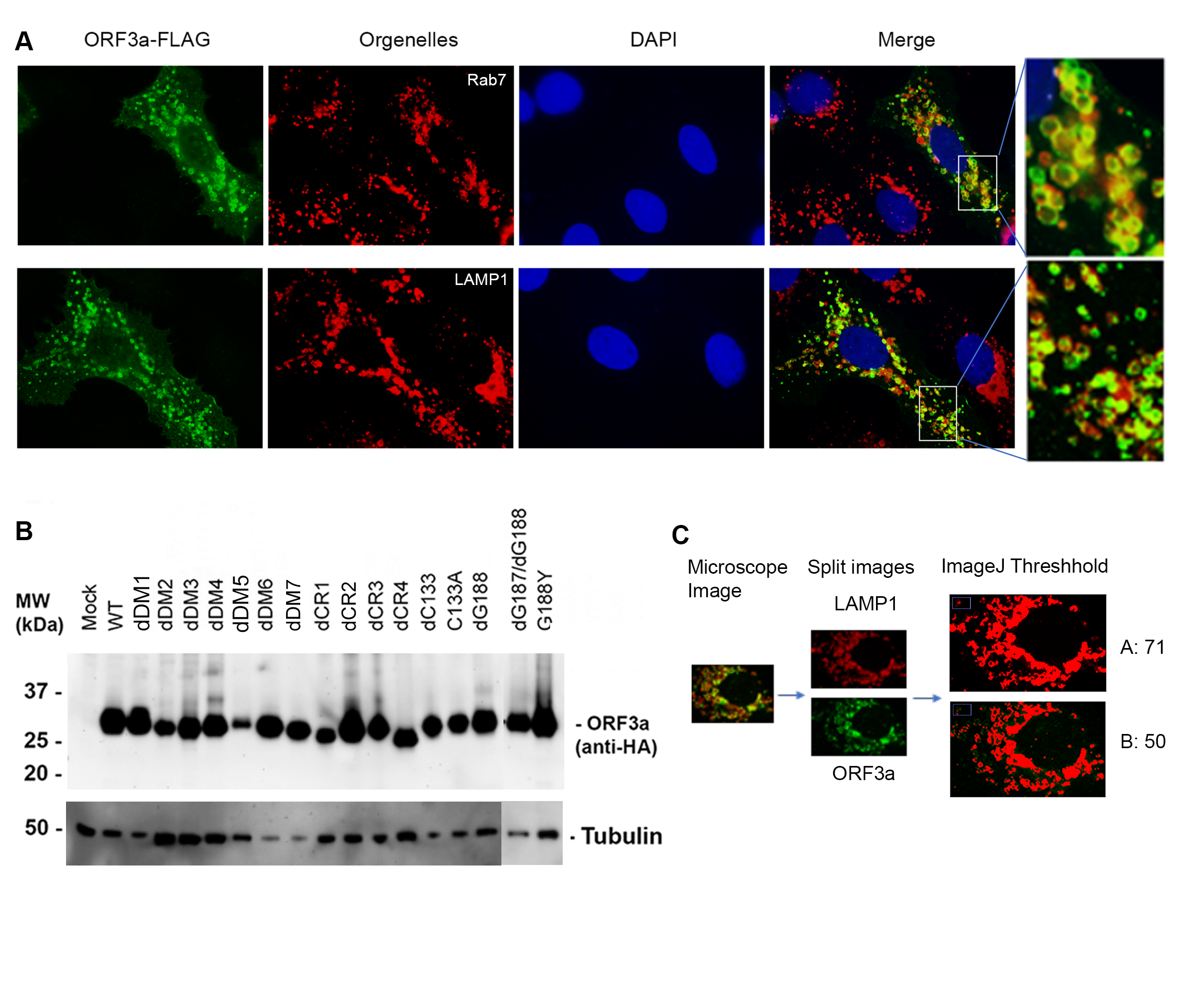

Supplement: Supplementary file 5 [file Image1.TIF]
